# The Warmer the Weather, the More Gram-Negative Bacteria - Impact of Temperature on Clinical Isolates in Intensive Care Units

**DOI:** 10.1371/journal.pone.0091105

**Published:** 2014-03-05

**Authors:** Frank Schwab, Petra Gastmeier, Elisabeth Meyer

**Affiliations:** 1 Institute of Hygiene and Environmental Medicine, Charité - University Medicine Berlin, Berlin, Germany; 2 National Reference Centre for Surveillance of Nosocomial Infections, Berlin, Germany; Amphia Ziekenhuis, Netherlands

## Abstract

**Background:**

We investigated the relationship between average monthly temperature and the most common clinical pathogens causing infections in intensive care patients.

**Methods:**

A prospective unit-based study in 73 German intensive care units located in 41 different hospitals and 31 different cities with total 188,949 pathogen isolates (102,377 Gram-positives and 86,572 Gram-negatives) from 2001 to 2012. We estimated the relationship between the number of clinical pathogens per month and the average temperature in the month of isolation and in the month prior to isolation while adjusting for confounders and long-term trends using time series analysis. Adjusted incidence rate ratios for temperature parameters were estimated based on generalized estimating equation models which account for clustering effects.

**Results:**

The incidence density of Gram-negative pathogens was 15% (IRR 1.15, 95%CI 1.10–1.21) higher at temperatures ≥20°C than at temperatures below 5°C. E. cloacae occurred 43% (IRR = 1.43; 95%CI 1.31–1.56) more frequently at high temperatures, A. baumannii 37% (IRR = 1.37; 95%CI 1.11–1.69), S. maltophilia 32% (IRR = 1.32; 95%CI 1.12–1.57), K. pneumoniae 26% (IRR = 1.26; 95%CI 1.13–1.39), Citrobacter spp. 19% (IRR = 1.19; 95%CI 0.99–1.44) and coagulase-negative staphylococci 13% (IRR = 1.13; 95%CI 1.04–1.22). By contrast, S. pneumoniae 35% (IRR = 0.65; 95%CI 0.50–0.84) less frequently isolated at high temperatures. For each 5°C increase, we observed a 3% (IRR = 1.03; 95%CI 1.02–1.04) increase of Gram-negative pathogens. This increase was highest for A. baumannii with 8% (IRR = 1.08; 95%CI 1.05–1.12) followed by K. pneumoniae, Citrobacter spp. and E. cloacae with 7%.

**Conclusion:**

Clinical pathogens vary by incidence density with temperature. Significant higher incidence densities of Gram-negative pathogens were observed during summer whereas S. pneumoniae peaked in winter. There is increasing evidence that different seasonality due to physiologic changes underlies host susceptibility to different bacterial pathogens. Even if the underlying mechanisms are not yet clear, the temperature-dependent seasonality of pathogens has implications for infection control and study design.

## Introduction

Seasonality is characteristic of many infectious diseases and has been recognized since Hippocrates who began his first book *Epidemics* with the description of the weather [1]. He wrote in 400 BC: “Whoever wishes to investigate medicine properly should proceed thus: in the first place to consider the seasons of the year…”.

A strong seasonal effect can be seen in many respiratory or bacterial gastrointestinal infections and in seasonally recurring childhood infections [2], [3]. Respiratory infections follow seasonal patterns. In temperate climates, respiratory illness is most common in winter months, and in tropical settings the incidence of lower respiratory-tract infections is generally increased during the rainy season [4].

By contrast, *Acinetobacter* infections show infection rates twice as high in late summer months as in early winter months [5]. Data on summer peaks in the incidences of other Gram-negative bacterial infections among hospitalized patients have only recently been published [6], [7], [8]. The authors of the study by the University of Maryland reported that incidence rates for *E. coli* were 12 percent higher in summer than in winter. Rates for *E. cloacae* were even 46 percent higher with the same seasonal variation. Seasonal variation has been described also for non-infectious diseases like depression, heart attack or stroke [9], [10].

Multicenter data on the seasonality of Gram-positive and Gram-negative clinical isolates in ICUs remain scare [11], [12]. Thus, the aim of our study was to look for temperature associations of pathogens in a network of geographically variant intensive care units in Germany and to discuss reasons for and implications of seasonal variation.

## Materials and Methods

### Ethics Statement

All data were anonymous and collected in accordance with the German recommendation for good epidemiological practice with respect to data protection [13]. As a federal law, the German Protection against Infection Act (Infektionsschutzgesetz §23) regulates the prevention and management of infectious disease in humans. All hospitals are obliged to continuously collect and analyse continuously healthcare-associated infections and drug-resistant pathogens [14]. These data are reported regularly to the National Reference Centre for the Surveillance for Nosocomial Infections. Ethical approval and informed consent were therefore not required.

### Intensive Care Units

From January 2001 to December 2012, a total of 73 German adult intensive care units (ICU) reported data to SARI (Surveillance of Antimicrobial Use and Antimicrobial Resistance in Intensive Care Units) [15]. The ICUs are located in 41 different hospitals and 34 different cities in Germany.

Data for air-conditioning were available for 64 of the 73 ICUs (88%). Thereof, 83% were air-conditioned during the entire analysis period, 6% were air-conditioned during parts of the analysis period and 11% were not air-conditioned.

### Data Collection

Monthly susceptibility data were collected from microbiology laboratories for 5 Gram-positive pathogens (*S. aureus*, *Coagulase negative staphylococci* (CoNS), *E. faecalis and faecium, S. pneumoniae*) and 8 Gram-negative pathogens (*E. coli, P. aeruginosa, K. pneumoniae, E. cloacae, S. maltophilia, S. marcescens, Citrobacter spp., A. baumannii*). Copy strains - defined as an isolate of the same species showing the same susceptibility pattern throughout the period of one month in the same patient, no matter what the site of isolation - were excluded. During data collection, it was not possible to differentiate culture data from isolates obtained less than or equal to 48 hours after ICU or hospital admission from isolates obtained greater than 48 hours (nosocomial). Monthly patient and device days were collected from ICU personal. Temperature data were collected as means per month in each individual year for cities or states from the publicly available database of the *Deutscher Wetterdienst* [16].

### Statistical Analysis

The incidence rate of isolates (IR) was defined as being the number of isolates of a pathogen per 1000 patient days. We investigated the following temperature parameters:

1. temperature in the month of isolation (categorized by 5 degree Celsius (°C) steps) to investigate the association with temperature including the proportionality (linearity) of the association;
2. temperature in the month prior to isolation (categorized by 5°C steps) to investigate the temporal shift/delay in the association (to reflect endogenous colonisation), as well as the proportionality of the association.

Incidence rates with 95% confidence intervals were calculated for each pathogen for the 2 above-mentioned parameters.

Time series analysis using generalized linear models were performed to estimate the association of the number of pathogens per month with temperature parameters.

Since observations within an ICU are not statistically independent due to the diagnostic policies (especially the frequency of microbiological tests), adjusted incidence rate ratios (IRR) with 95% confidence intervals (CI) were estimated. They were based on generalized estimating equation (GEE) models which account for this clustering effect by using an exchangeable correlation structure [17].

We used negative binomial distribution in the models instead of Poisson distribution because the variance exceeds the mean and because overdispersion was observed for all type of pathogens. The Lagrange multiplier test was used to test whether the negative binomial model significantly differs from the Poisson model. The log number of patient days during each month was treated as an offset in the model.

The following parameters considered confounding in the frequency of the occurrence of pathogens: long-term trends between 2001 and 2012 (linear, quadratic and cubic time trend), the number of pathogens in the previous month as an autoregressive factor (AR1), and the following structural parameters: type of ICU (medical, surgical, interdisciplinary), size of ICU (</> = 12 beds), type of hospital in which ICU is located (university, teaching, other) and size of hospital in which ICU is located (</> = 1000 beds). All confounding parameters were parameterized as continuous or dummy parameters and added 1 degree of freedom to the model.

### The Multivariable Model Building Strategy was Performed in Two Steps for each Pathogen

1. All confounding parameters were included in a full GEE model, and non-significant parameters were excluded stepwise. The selection criterion was the smallest Chi-square value and p> = 0.05 in the type III score statistic.
2. For each pathogen, a GEE model was calculated with the significant confounding parameters from the first step and the temperature parameters. Temperature parameters were considered the categorical variable in the model with four degrees of freedom in order to test differences in the frequency of pathogens between temperature intervals; temperature intervals were considered the ordinal/linear covariate in the model with one degree of freedom in order to test the linearity of the association.

An additional analysis was performed with the following monthly process parameters: length of stay (LOS, days); and device use (device days per 100 patient days) for central venous catheters, urinary tract catheters and ventilators to adjust for the severity of illness. This additional analysis showed the same significant results as without these parameters. However, the process parameters were only available in 85 percent of months analyzed. Therefore, we present our results without adjusting for LOS and device use.

The quasi-likelihood information criterion (QIC) as a modification of the Akaike information criterion (ACI) was used as goodness-of-fit measure in the GEE model. P-values less than 0.05 were considered significant. All analyses were performed using SPSS [IBM SPSS statistics, Somer, NY, USA] and SAS [SAS Institute, Cary, NC, USA].

## Results

73 ICUs reported data on 2,243,856 patient days (pd) and 188,949 pathogen isolates (102,377 Gram-positives and 86,572 Gram-negatives). Table 1 shows the characteristics of the 73 ICUs.

**Table 1:** Baseline characteristics of 73 German ICUs, January 2001 to December 2012.

Parameters	Statistic
Structure parameters of ICU and hospital	
Number ICUs (number hospitals; number cities)	73 (41; 34)
Type of ICU	
interdisciplinary, No. (%)	34 (46.6)
medical, No. (%)	18 (24.7)
surgical, No. (%)	21 (28.8)
Type of hospital in which ICU is located	
university, No. (%)	23 (31.5)
teaching, No. (%)	40 (54.8)
other, No. (%)	10 (13.7)
Size of ICU (No. beds), median (IQR)	12 (10–18)
Size of hospital in which ICU is located (No. beds), median (IQR)	854 (488–1229)
Analyzed months	
months total, No.	5965
Months per ICU^a^, Median (IQR)	84 (36–127)
Distribution of temperature (°C)	
Monthly temperature, median (IQR); (Min; Max)	9.9 (4.4–16.2); (−5.6; 24.4)
Process parameters^a^	
Incidence rate of pathogens^b^ (No. pathogens/1000 patient days), median (IQR)	78 (61–97)
Length of stay and device use (N = 62 ICUs)	
Length of stay (days), median (IQR)	4.0 (3.2–5.5)
Ventilation utilization (No. ventilation days/100 patient days), median (IQR)	43 (31–57)
Central venous catheter utilization (No. central venous catheter -days/100 patient days), median (IQR)	74 (57–84)
Urinary tract catheter utilization (No. urinary tract catheter-days/100 patient days), median (IQR)	85 (76–91)

Abbreviations: No., number; ICU, intensive care unit; IQR, interquartile range; °C, Degree Celsius.

a calculated per ICU.

b considered 5 Gram-positive pathogens (*S. aureus*, *Coagulase negative staphylococci, E. faecalis and faecium, S. pneumoniae*) and 8 Gram-negative pathogens (*E. coli, P. aeruginosa, K. pneumoniae, E. cloacae, S. maltophilia, S. marcescens, Citrobacter spp., A. baumannii*); Copy strains were excluded.

In median, 84 months per ICU and 78 pathogens per 1000 patient days were analysed from January 2001 to December 2012. The median monthly temperature was 9.9°C and varied between −5.6 and 24.4°C.

**Figure 1:**
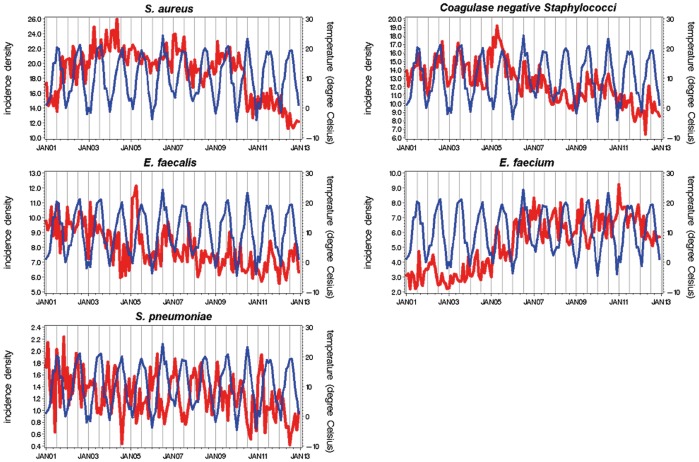
Time series of monthly aggregated incidence densities of Gram-positive pathogens (bold red line) and temperature (thin blue line) in 73 German ICUs, January 2001 to December 2012.

**Figure 2:**
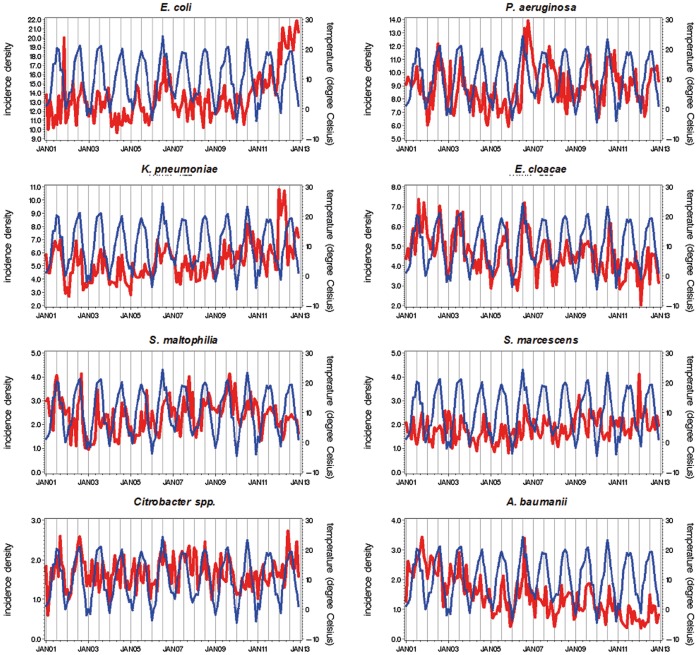
Time series of monthly aggregated incidence density of Gram-negative pathogens (bold red line) and temperature (thin blue line) in 73 German ICUs, January 2001 to December 2012.

The time series of the incidence rates for the different Gram-positive and Gram-negative pathogens in 73 German ICUs and mean monthly temperature in Germany are depicted in figure 1 and figure 2. Generally, the incidence of the Gram-negatives follows the increase in the temperature and follows a decrease in temperature likewise. This is not the case for Gram-positives like. *S. pneumoniae* or *S. aureus*.

Table 2 gives the number and the incidence rate of pathogens by temperature in the month of isolation over the 12-year period, stratified by 5°C temperature interval steps.

**Table 2:** Incidence rate (IR) for Gram-positive and Gram-negative pathogens by temperature in the month of isolation. Data are based on in 73 German ICUs, January 2001 to December 2012.

Parameters	Total			Temperature (mean) in the month of isolation									
				<5°C		5–<10°C		10–<15°C		15–<20°C		> = 20°C	
analysed months, No. (%)	5965 (100)			1693 (28.4)		1323 (22.2)		1019 (17.1)		1658 (27.8)		271 (4.6)	
Pathogens	No.	IR	95%CI	IR	95% CI	IR	95% CI	IR	95% CI	IR	95% CI	IR	95% CI
All^a^	188,949	84.2	84.0; 84.6	85.1	84.0; 84.6	81.2	80.4; 82.0	84.6	83.6; 85.5	84.9	84.2; 85.6	88.1	86.3; 90.0
All GP^a^	102,377	45.6	45.3; 45.9	47.9	45.3; 45.9	43.1	42.5; 43.7	45.8	45.1; 46.5	45.2	44.7; 45.8	45.5	44.2; 46.8
* S. aureus*	42,085	18.8	18.6; 18.9	20.3	18.6; 18.9	17.1	16.8; 17.5	19.1	18.6; 19.5	18.5	18.2; 18.9	17.4	16.6; 18.3
* CoNS*	27,654	12.3	12.2; 12.5	12.4	12.2; 12.5	11.3	11.0; 11.6	12.4	12.1; 12.8	12.7	12.5; 13.0	13.8	13.1; 14.6
* E. faecalis*	17,595	7.8	7.7; 8.0	8.0	7.7; 8.0	7.8	7.5; 8.0	7.8	7.5; 8.1	7.7	7.5; 7.9	8.1	7.6; 8.7
* E. faecium*	12,250	5.5	5.4; 5.6	5.7	5.4; 5.6	5.6	5.4; 5.8	5.4	5.2; 5.7	5.2	5.0; 5.3	5.2	4.8; 5.7
* S. pneumoniae*	2,793	1.2	1.2; 1.3	1.5	1.2; 1.3	1.3	1.2; 1.4	1.1	1.0; 1.2	1.1	1.0; 1.2	0.9	0.8; 1.2
All GN^a^	86,572	38.6	38.3; 38.8	37.2	38.3; 38.8	38.1	37.6; 38.7	38.7	38.1; 39.4	39.7	39.2; 40.2	42.6	41.3; 43.9
* E. coli*	29,369	13.1	12.9; 13.2	13.0	12.9; 13.2	13.2	12.9; 13.6	12.9	12.5; 13.2	13.2	12.9; 13.5	13.0	12.3; 13.8
* P. aeruginosa*	19,849	8.8	8.7; 9.0	8.5	8.7; 9.0	9.1	8.8; 9.4	8.7	8.4; 9.0	9.0	8.8; 9.2	10.0	9.3; 10.6
* K. pneumoniae*	11,394	5.1	5.0; 5.2	4.8	5.0; 5.2	4.9	4.7; 5.1	5.2	5.0; 5.4	5.3	5.1; 5.5	6.0	5.5; 6.5
* E. cloacae*	9,935	4.4	4.3; 4.5	4.1	4.3; 4.5	4.1	4.0; 4.3	4.6	4.4; 4.8	4.7	4.6; 4.9	5.6	5.1; 6.1
* S. maltophilia*	5,262	2.3	2.3; 2.4	2.3	2.3; 2.4	2.1	2.0; 2.2	2.4	2.3; 2.6	2.5	2.3; 2.6	2.9	2.5; 3.2
* S. marcescens*	4,061	1.8	1.8; 1.9	1.9	1.8; 1.9	1.8	1.7; 1.9	1.8	1.7; 2.0	1.7	1.6; 1.8	1.7	1.4; 2.0
* Citrobacter. spp.*	3,635	1.6	1.6; 1.7	1.5	1.6; 1.7	1.5	1.4; 1.7	1.7	1.6; 1.9	1.8	1.7; 1.9	1.7	1.4; 2.0
* A. baumannii*	3,067	1.4	1.3; 1.4	1.2	1.3; 1.4	1.3	1.2; 1.4	1.4	1.3; 1.5	1.5	1.4; 1.6	1.8	1.6; 2.1

Abbreviations: °C, degrees Celsius; No., number; IR, incidence rate defined by No. pathogens per 1000 patient days; CI, confidence interval; GP, Gram-positive pathogens; GN, Gram-negative pathogens; CoNS, coagulase negative Staphylococci.

a considered 5 Gram-positive pathogens (*S. aureus*, *Coagulase negative staphylococci, E. faecalis and faecium, S. pneumoniae*) and/or 8 Gram-negative pathogens (*E. coli, P. aeruginosa, K. pneumoniae, E. cloacae, S. maltophilia, S. marcescens, Citrobacter spp., A. baumannii*); copy strains were excluded.

### Temperature in the Month of Isolation

**Figure 3:**
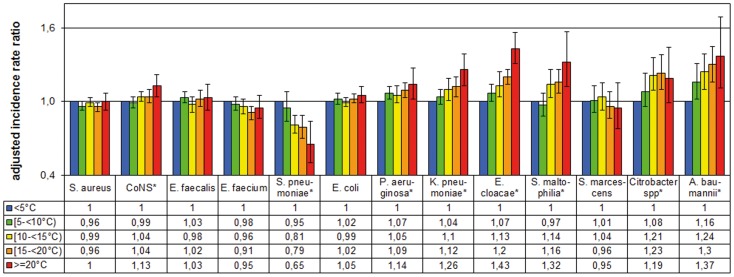
Adjusted incidence rate ratios for Gram-positive and Gram-negative pathogens by temperature in the month of isolation. As reference we defined temperatures in the month of isolation <5°C. For the calculation we used generalized estimating equations (GEE) models using negative binomial distribution and logarithmic transformed patient days as offset parameter. Data based on 5965 monthly data sets of 73 different German ICUs, January 2001 to December 2012. The adjusted models accounts for cluster effects within an ICU and for significant confounding parameters from the first model building step: *S.aureus* ^(a, d, f)^, coagulase negative *staphylococci* ^(a, b)^, *E. faecalis* ^(b)^, *E. faecium* ^(a, c, d, e)^, *S. pneumoniae* ^(a, b)^, *E. coli* ^(e)^, *P. aeruginosa* ^(a, c)^, *K. pneumoniae* ^(no confounder)^, *E. cloacae* ^(a, b, c, d)^, *S. maltophilia* ^(a, b, c, f)^, *S. marcescens* ^(a, f)^, *Citrobacter* spp. ^(no confounder)^, *A. baumannii* ^(a, b, f, g)^ in which: ^a^, autoregressive factor AR(1) number pathogens in the previous month; ^b^, linear time trend; ^c^, quadratic time trend; ^d^, cubic time trend; ^e^, ICU is located in a university hospital; ^f^, hospital size > = 1000 beds; ^g^, type of ICU medical; ^h^, type of ICU surgical. *P<0.05 in chi-square test with 4 degrees of freedom (type III test) in the adjusted GEE model; Whiskers represent 95% confidence intervals.

The adjusted incidence rate ratios (IRR) of the temperature in the month of isolation are shown in figure 3 and in more detail in table 3.

**Table 3:** Adjusted incidence rate ratios for Gram-positive and Gram-negative pathogens by temperature in the month of isolation. As reference we defined temperatures in the month of isolation <5°C.

Pathogens	Temperature in the month of isolation									per 5°C increase			
	5–<10°C		10–<15°C		15–<20°C		> = 20°C		p-value*				p-value**
	IRR	95% CI	IRR	95% CI	IRR	95% CI	IRR	95% CI		IRR	95%CI	p	
All	0.99	0.96; 1.02	1.02	0.99; 1.05	1.02	0.99; 1.05	1.07	1.02; 1.11	0.009	1.01	1.00; 1.02	0.008	0.006
All GP^a^^,^ d	0.98	0.96; 1.01	0.99	0.96; 1.02	0.98	0.95; 1.01	1.02	0.97; 1.06	0.548	1.00	0.99; 1.00	0.418	0.409
* S. aureus* a ^,^ d ^,^ f	0.96	0.93; 1.00	0.99	0.96; 1.03	0.96	0.92; 0.99	1.00	0.93; 1.07	0.090	0.99	0.98; 1.00	0.044	0.049
* CoNS* a ^,^ b	0.99	0.95; 1.04	1.04	1.00; 1.08	1.04	0.99; 1.10	1.13	1.04; 1.22	0.007	1.02	1.01; 1.04	0.002	0.004
* E. faecalis* b	1.03	0.99; 1.08	0.98	0.91; 1.04	1.02	0.96; 1.09	1.03	0.93; 1.14	0.250	1.00	0.98; 1.02	0.757	0.754
* E. faecium* a ^,^ c ^,^ d ^,^ e	0.98	0.93; 1.04	0.96	0.90; 1.02	0.91	0.85; 0.96	0.95	0.86; 1.05	0.063	0.97	0.96; 0.99	0.002	0.004
* S. pneumoniae* a ^,^ b	0.95	0.84; 1.08	0.81	0.74; 0.89	0.79	0.70; 0.89	0.65	0.50; 0.84	0.001	0.91	0.88; 0.94	<0.001	0.001
All GN	1.04	1.02; 1.07	1.06	1.03; 1.10	1.08	1.05; 1.12	1.15	1.10; 1.21	<0.001	1.03	1.02; 1.04	<0.001	<0.001
* E. coli e*	1.02	0.98; 1.07	0.99	0.96; 1.03	1.02	0.99; 1.06	1.05	0.99; 1.12	0.329	1.01	1.00; 1.02	0.201	0.203
* P. aeruginosa* a ^,^ c	1.07	1.02; 1.12	1.05	0.99; 1.13	1.09	1.03; 1.15	1.14	1.02; 1.27	0.011	1.03	1.01; 1.04	0.002	0.003
* K. pneumoniae*	1.04	0.98; 1.10	1.10	1.01; 1.19	1.12	1.04; 1.20	1.26	1.13; 1.39	0.008	1.05	1.02; 1.07	<0.001	0.000
* E. cloacae* a ^,^ b ^,^ c ^,^ d	1.07	1.00; 1.14	1.13	1.04; 1.24	1.20	1.14; 1.26	1.43	1.31; 1.56	<0.001	1.07	1.05; 1.09	<0.001	<0.001
* S. maltophilia* a ^,^ b ^,^ c ^,^ f	0.97	0.88; 1.07	1.14	1.03; 1.26	1.16	1.07; 1.26	1.32	1.12; 1.57	0.001	1.07	1.04; 1.09	<0.001	<0.001
* S. marcescens* a ^,^ f	1.01	0.91; 1.13	1.04	0.93; 1.15	0.96	0.86; 1.08	0.95	0.78; 1.15	0.072	0.99	0.96; 1.02	0.451	0.449
* Citrobacter spp.*	1.08	0.96; 1.23	1.21	1.09; 1.36	1.23	1.10; 1.38	1.19	0.99; 1.44	0.006	1.07	1.03; 1.10	<0.001	<0.001
* A. baumanii* a ^,^ b ^,^ f ^,^ g	1.16	1.02; 1.31	1.24	1.10; 1.39	1.30	1.16; 1.45	1.37	1.11; 1.69	0.002	1.08	1.05; 1.12	<0.001	<0.001

Abbreviations: °C, degree Celsius; IRR, incidence rate ratio; CI, confidence interval; GP, Gram-positive pathogens; GN, Gram-negative pathogens; CoNS, *coagulase negative Staphylococci*.

*P-value, chi-square test with 4 degree of freedom (type III test);

**P-value, chi-square test with 1 degree of freedom (type III test);

NOTE: Adjusted incidence rate ratios were calculated using generalized estimating equations models using negative binomial distribution and logarithmic transformed patient days as offset parameter; 5965 monthly data sets of 73 different ICUs, January 2001 to December 2012. The adjusted models accounts for cluster effects within an ICU and for significant confounding parameters from the first model building step:

a , autoregressive factor AR(1) number pathogens in the previous month;

b , linear time trend;

c , quadratic time trend;

d , cubic time trend;

e , ICU is located in a university hospital;

f , hospital size > = 1000 beds;

g , type of ICU medical;

h , type of ICU surgical.

In total, the incidence density of Gram-negatives pathogens was 15% (IRR 1.15, 95%CI 1.10–1.21) higher in months with high temperatures (≥20°C) when compared to months with low temperatures (<5°C).

In eight of the thirteen pathogens (two of the five Gram-positives; six of the eight Gram-negatives), the adjusted IRR for temperature in the current month was significant (type III test). In *S. pneumoniae,* the IRR was 0.65 (95%CI 0.50–0.84) for months ≥20°C compared to months <5°C. In other words, *S. pneumoniae* occurred 35 percent less frequently at high temperatures (≥20°C) than at low temperatures (<5°C).

Figure 1 shows that the incidence density of *S. pneumoniae* and the temperature were in opposite phases: when it was cold, the incidence of *S. pneumoniae* was high.

The other seven pathogens were significantly positively associated with high temperatures (≥20°C). *E. cloacae* occurred 43% (IRR = 1.43; 95%CI 1.31–1.56) more frequently at high temperatures, *A. baumannii* 37% (IRR = 1.37; 95%CI 1.11–1.69), *S. maltophilia* 32% (IRR = 1.32; 95%CI 1.12–1.57), *K. pneumoniae* 26% (IRR = 1.26; 95%CI 1.13–1.39), *Citrobacter spp.* 19% (IRR = 1.19; 95%CI 0.99–1.44) and coagulase-negative staphylococci 13% (IRR = 1.13; 95%CI 1.04–1.22).

### *Linearity*

Testing the linearity of the relationship (testing temperature parameters as ordinal/linear covariate in the model with 1 degree of freedom) shows that the relationship was linear (figure 3, table 3). For the Gram-negative pathogens, the IRR was 1.03 (95%CI 1.02–1.04) per 5°C step. For each 5°C increase, we observed a 3% increase in the incidence density of Gram-negative pathogens. Specifically, there was an 8% increase for *A. baumannii* (IRR = 1.08; 95%CI 1.05–1.12), followed by *K. pneumoniae*, *Citrobacter spp*. and *E. cloacae* with 7%. There was a 9% decrease for *S. pneumoniae* (IRR = 0.91; 95%CI 95%CI 0.88–0.94).

### Temperature in the Month Prior to Isolation

To account for the temporal shift between the temperature and the frequency of pathogens, we considered the temperature in the month prior to isolation. Table 4 gives the number and the incidence density of pathogens over the 12-year period, stratified by 5°C steps temperature intervals in the month prior to isolation.

**Table 4:** Incidence rates for Gram-positive and Gram-negative pathogens by temperature in the month prior to isolation. Data based on 73 German ICUs, January 2001 to December 2012.

Parameters	Total			Temperature (mean) in the month prior to isoaltion									
				<5°C		5–<10°C		10–<15°C		15–<20°C		> = 20°C	
analysed months,No. (%)	5965 (100)			1698 (28.5)		1323 (22.2)		1018 (17.1)		1654 (27.7)		272 (4.6)	
Pathogens	No.	IR	95%CI	IR	95% CI	IR	95% CI	IR	95% CI	IR	95% CI	IR	95% CI
All^a^	188,949	84.2	84.0; 84.6	85.6	84.9; 86.3	80.0	79.2; 80.7	82.8	81.9; 83.8	86.2	85.5; 86.9	89.7	87.8; 91.5
All GP^a^	102,377	45.6	45.3; 45.9	48.7	48.2; 49.3	43.2	42.6; 43.8	44.4	43.8; 45.1	45.3	44.7; 45.8	44.6	43.3; 45.9
* S. aureus*	42,085	18.8	18.6; 18.9	20.6	20.3; 21.0	17.3	16.9; 17.6	18.2	17.8; 18.6	18.6	18.3; 19.0	17.1	16.3; 17.9
* CoNS*	27,654	12.3	12.2; 12.5	12.5	12.2; 12.7	11.7	11.4; 12.0	12.1	11.7; 12.4	12.7	12.4; 13.0	13.0	12.3; 13.8
* E. faecalis*	17,595	7.8	7.7; 8.0	8.2	8.0; 8.4	7.4	7.2; 7.6	7.8	7.5; 8.1	7.8	7.6; 8.0	8.3	7.7; 8.9
* E. faecium*	12,250	5.5	5.4; 5.6	5.9	5.7; 6.1	5.6	5.4; 5.8	5.2	5.0; 5.5	5.1	4.9; 5.3	5.2	4.8; 5.7
* S. pneumoniae*	2,793	1.2	1.2; 1.3	1.5	1.4; 1.6	1.2	1.1; 1.3	1.2	1.1; 1.3	1.1	1.0; 1.1	0.9	0.8; 1.2
All GN^a^	86,572	38.6	38.3; 38.8	36.8	36.4; 37.3	36.8	36.2; 37.3	38.4	37.8; 39.0	40.9	40.4; 41.4	45.1	43.8; 46.4
* E. coli*	29,369	13.1	12.9; 13.2	13.1	12.8; 13.4	12.7	12.4; 13.0	13.2	12.8; 13.6	13.1	12.9; 13.4	14.2	13.5; 15.0
* P. aeruginosa*	19,849	8.8	8.7; 9.0	8.4	8.2; 8.6	8.5	8.2; 8.7	8.7	8.4; 9.0	9.4	9.1; 9.6	10.6	9.9; 11.2
* K. pneumoniae*	11,394	5.1	5.0; 5.2	4.7	4.5; 4.9	4.9	4.7; 5.1	5.0	4.8; 5.2	5.5	5.3; 5.7	5.9	5.4; 6.4
* E. cloacae*	9,935	4.4	4.3; 4.5	4.0	3.8; 4.1	4.0	3.9; 4.2	4.5	4.3; 4.7	5.0	4.8; 5.1	5.9	5.4; 6.4
* S. maltophilia*	5,262	2.3	2.3; 2.4	2.1	2.0; 2.2	2.0	1.9; 2.2	2.4	2.2; 2.5	2.8	2.7; 2.9	2.7	2.4; 3.0
* S. marcescens*	4,061	1.8	1.8; 1.9	1.9	1.7; 2.0	1.8	1.7; 1.9	1.7	1.5; 1.8	1.8	1.7; 1.9	1.9	1.6; 2.2
* Citrobacter. spp.*	3,635	1.6	1.6; 1.7	1.5	1.4; 1.6	1.5	1.4; 1.6	1.7	1.5; 1.8	1.8	1.7; 1.9	1.9	1.7; 2.2
* A. baumannii*	3,067	1.4	1.3; 1.4	1.2	1.1; 1.3	1.3	1.2; 1.4	1.3	1.2; 1.4	1.5	1.4; 1.6	2.0	1.8; 2.4

Abbreviations: °C, degrees Celsius; No., number; IR, incidence rate defined by No. pathogens per 1000 patient days; CI, confidence interval; GP, Gram-positive pathogens; GN, Gram-negative pathogens; CoNS, coagulase negative Staphylococci.

a considered 5 Gram-positive pathogens (*S. aureus*, *Coagulase negative staphylococci, E. faecalis and faecium, S. pneumoniae*) and/or 8 Gram-negative pathogens (*E. coli, P. aeruginosa, K. pneumoniae, E. cloacae, S. maltophilia, S. marcescens, Citrobacter spp., A. baumannii*); copy strains were excluded.

**Figure 4:**
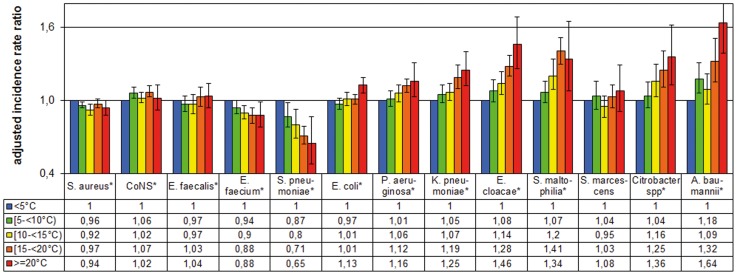
Adjusted incidence rate ratios for Gram-positive and Gram-negative pathogens by temperature in the month prior to isolation. As reference we defined temperatures in the month prior to isolation <5°C. For the calculation we used generalized estimating equations (GEE) models using negative binomial distribution and logarithmic transformed patient days as offset parameter. Data based on 5965 monthly data sets of 73 different German ICUs, January 2001 to December 2012. The adjusted models accounts for cluster effects within an ICU and for significant confounding parameters from the first model building step: *S.aureus* ^(a, d, f)^, coagulase negative *staphylococci* ^(a, b)^, *E. faecalis* ^(b)^, *E. faecium* ^(a, c, d, e)^, *S. pneumoniae* ^(a, b)^, *E. coli* ^(e)^, *P. aeruginosa* ^(a, c)^, *K. pneumoniae* ^(no confounder)^, *E. cloacae* ^(a, b, c, d)^, *S. maltophilia* ^(a, b, c, f)^, *S. marcescens* ^(a, f)^, *Citrobacter* spp. ^(no confounder)^, *A. baumannii* ^(a, b, f, g)^ in which: ^a^, autoregressive factor AR(1) number pathogens in the previous month; ^b^, linear time trend; ^c^, quadratic time trend; ^d^, cubic time trend; ^e^, ICU is located in a university hospital; ^f^, hospital size > = 1000 beds; ^g^, type of ICU medical; ^h^, type of ICU surgical. *P<0.05 in chi-square test with 4 degrees of freedom (type III test) in the adjusted GEE model; Whiskers represent 95% confidence intervals.

The adjusted incidence rate ratios (IRR) for the temperature in the month prior to isolation are shown in figure 4 and in more detail in table 5.

**Table 5:** Adjusted incidence rate ratios for Gram-positive and Gram-negative pathogens by temperature in the month prior to isolation. As reference we defined temperatures in the month prior to isolation <5°C.

Pathogens	Temperature in the month prior to isolation									per 5°C increase			
	5–<10°C		10–<15°C		15–<20°C		> = 20°C		p-value*				p-value**
	IRR	95%CI	IRR	95%CI	IRR	95%CI	IRR	95%CI		IRR	95%CI	p	
All	0.97	0.94; 1.00	0.99	0.96; 1.02	1.03	1.00; 1.07	1.08	1.02; 1.13	0.000	1.01	1.01; 1.02	0.002	0.002
All GP^a^^,^ d	0.98	0.96; 1.01	0.95	0.93; 0.98	0.99	0.97; 1.02	0.96	0.91; 1.01	0.057	0.99	0.99; 1.00	0.135	0.136
* S. aureus* a ^,^ d ^,^ f	0.96	0.94; 0.99	0.92	0.88; 0.97	0.97	0.94; 1.01	0.94	0.88; 1.00	0.015	0.99	0.98; 1.00	0.030	0.037
* CoNS* a ^,^ b	1.06	1.02; 1.11	1.02	0.98; 1.07	1.07	1.03; 1.12	1.02	0.92; 1.13	0.028	1.01	1.00; 1.03	0.020	0.025
* E. faecalis* b	0.97	0.91; 1.04	0.97	0.89; 1.05	1.03	0.95; 1.11	1.04	0.94; 1.14	0.059	1.01	0.99; 1.03	0.381	0.364
* E. faecium* a ^,^ c ^,^ d ^,^ e	0.94	0.89; 1.00	0.90	0.85; 0.96	0.88	0.81; 0.94	0.88	0.78; 0.99	0.009	0.96	0.94; 0.98	<0.001	0.001
* S. pneumoniae* a ^,^ b	0.87	0.78; 0.98	0.80	0.69; 0.93	0.71	0.64; 0.79	0.65	0.48; 0.87	0.000	0.89	0.86; 0.93	<0.001	<0.001
All GN	1.02	0.99; 1.05	1.06	1.03; 1.09	1.13	1.08; 1.17	1.23	1.16; 1.30	0.020	1.04	1.03; 1.06	<0.001	0.018
* E. coli e*	0.97	0.93; 1.02	1.01	0.96; 1.07	1.01	0.97; 1.05	1.13	1.06; 1.19	<0.001	1.01	1.00; 1.03	0.016	<0.001
* P. aeruginosa* a ^,^ c	1.01	0.95; 1.08	1.06	0.99; 1.13	1.12	1.07; 1.18	1.16	1.03; 1.31	0.003	1.04	1.02; 1.06	<0.001	<0.001
* K. pneumoniae*	1.05	0.98; 1.13	1.07	1.00; 1.14	1.19	1.10; 1.29	1.25	1.12; 1.40	0.003	1.06	1.03; 1.08	<0.001	<0.001
* E. cloacae* a ^,^ b ^,^ c ^,^ d	1.08	0.99; 1.17	1.14	1.05; 1.24	1.28	1.20; 1.37	1.46	1.26; 1.69	<0.001	1.09	1.07; 1.11	<0.001	<0.001
* S. maltophilia* a ^,^ b ^,^ c ^,^ f	1.07	0.98; 1.16	1.20	1.09; 1.34	1.41	1.30; 1.52	1.34	1.08; 1.65	<0.001	1.11	1.08; 1.14	<0.001	<0.001
* S. marcescens* a ^,^ f	1.04	0.93; 1.16	0.95	0.86; 1.04	1.03	0.94; 1.13	1.08	0.91; 1.29	0.410	1.01	0.98; 1.03	0.597	0.595
* Citrobacter spp.*	1.04	0.94; 1.15	1.16	1.03; 1.30	1.25	1.11; 1.41	1.36	1.13; 1.62	0.007	1.08	1.04; 1.12	<0.001	<0.001
* A. baumanii* a ^,^ b ^,^ f ^,^ g	1.18	1.06; 1.31	1.09	0.97; 1.22	1.32	1.15; 1.51	1.64	1.39; 1.94	<0.001	1.10	1.06; 1.13	<0.001	<0.001

Abbreviations: °C, degree Celsius; IRR, incidence rate ratio; CI, confidence interval; GP, Gram-positive pathogens; GN, Gram-negative pathogens; CoNS, *coagulase negative Staphylococci*.

* p-value, chi-square test with 4 degree of freedom (type III test); ** p-value, chi-square test with 1 degree of freedom (type III test);.

NOTE: Adjusted incidence rate ratios were calculated using generalized estimating equations models using negative binomial distribution and logarithmic transformed patient days as offset parameter; 5965 monthly data sets of 73 different ICUs, January 2001 to December 2012. The adjusted models accounts for cluster effects within an ICU and for significant confounding parameters from the first model building step:

a , autoregressive factor AR(1) number pathogens in the previous month;

b , linear time trend;

c , quadratic time trend;

d , cubic time trend;

e , ICU is located in a university hospital;

f , hospital size > = 1000 beds;

g , type of ICU medical;

h , type of ICU surgical.

In eleven of the thirteen pathogens, the adjusted IRR for temperature in the month prior to isolation was significant. For *S. aureus*, *E. faecium* and *S. pneumoniae,* the association was negative compared to month prior to isolation <5°C; for the seven Gram-negatives (excluding *S. marscecens*) and CoNS, it was positive. Moreover, testing the linear associations showed significance (excluding *E. faecalis* and *S. marscecens*). Moreover, the differences were more pronounced in comparison to the results which take the temperature in the month of isolation into account.

## Discussion

The four main findings of this study in 73 ICUs and on 188,949 non-duplicate bacterial cultures over a 12-year period are as follows:

- Eleven of the thirteen analysed pathogens were associated with temperature (*S. pneumoniae*, E. *coli, K. pneumoniae, E. cloacae, S. maltophilia, Citrobacter spp., A. baumannii*, CoNS; *S. aureus, E. faecium* and *P. aeruginosa*). Only *E. faecalis* and *S. marcescens* showed no temperature association.
- The Gram-positive pathogen CoNS and the Gram-negatives *E. coli, P. aeruginosa, K. pneumoniae, E. cloacae, S. maltophilia, Citrobacter spp., A. baumannii* were positively associated with temperature. In other words, the warmer the weather, the higher the incidence density. *S. aureus, E. faecium* and *S. pneumoniae* were negatively associated with temperature; i.e. the colder the weather, the higher the incidence density.
- The associations were proportional to the temperature intervals (or inversely proportional, respectively).
- The association of the frequency of isolation and temperature for Gram-negatives was stronger when the mean monthly temperature in the month prior to isolation was considered instead of the temperature in the month of isolation.

Explanations for these differences are mainly hypothetical. There is conflicting evidence in support of different ideas, suggesting that there is no one single reason but rather a collection of different factors. One reason could be that environmental factors like temperature, light, humidity and wind influence the frequency of pathogen isolation. However, room temperature and other environmental factors are usually fairly constant in ICUs, in hospitals and in laboratories. Therefore, it seems more likely that a variation in the endogenous flora of the patient plays a role.

Most infections, including nosocomial infections, are endogenous in nature. In a comprehensive study in German ICUs, only 14.5 percent of nosocomial infections were associated with exogenous patient-to-patient transmission [18]. The mostly endogenous origin of infection or colonisation could also explain why the association of frequency of occurrence with temperature a month before the isolation of the pathogen was the same or even more prominent than the association with the current outside temperature. This holds true for infections that are generally classified as community acquired, such as infections caused by *S. pneumoniae,* as well as those mostly classified as nosocomial like infections by *A. baumannii*. But irrespective of the endogenous origin, why do infections with some Gram-positive and Gram-negative bacteria peak in different seasons of the year?

The phenomenon that pneumococcal infections increase in winter has been repeatedly documented [19], [20]. The incidence density ratio of *S. pneumoniae* was 35 percent lower in month with temperatures > = 20°C (compared to months <5°C) in our study as well, which is just the opposite of what we found in Gram-negatives. Dowell *et al.* evaluated data of seven geographic areas in the United States and found that pneumococcal disease correlated inversely with temperature [19]. One might expect higher rates throughout the year if colder temperature is what drives the winter increase. Paradoxically, the coldest states had the lowest rates in their study. Dowell believes that the photoperiod (dark-light cycles) and not temperature per se contributes to seasonality. The incidence of infections may therefore be due to changes in the susceptibility of the host to the particular pathogen. Susceptibility might differ for different pathogens. It might depend on photoperiod being the most pervasive signal for seasonal changes in the biological system and may be mediated by the length of the daily melatonin pulse (melatonin, a pineal hormone, has been shown to mediate seasonal adjustments in immune function) and a range of other physiologic parameters like the serum cortisol level, IL-6, CD4 and CD8 cells or circulating B cells [21].

Other explanations could be pathogen appearance and disappearance or host behaviour changes like seasonal variation in the consumption of food or outdoor activities [21]. The periodic occurrence of gastrointestinal infections and outbreaks in summer is well recognized [22]. However, the objection can be made that the most prominent summer peak of Gram-negative clinical isolates was by *A. baumannii,* a pathogen which does not appear to be a typical environmental organism but has its main reservoir in humans. By contrast, other *Acinetobacter* species indeed seem to be distributed widely in nature. *A. calcoaceticus*, for example, is found in water and soil and on vegetables [23].

In a landmark study, Perencevich *et. al.* analyzed 26,624 unique clinical cultures over 8 years in a US-American university hospital and observed significant summer peaks in the incidences of non-duplicate clinical cultures of *E. coli*, *A. baumannii*, *E. cloacae* and *P. aeruginosa* [6]. This was true for Gram-negative but not for Gram-positive pathogens. Interestingly, the results of our study were very similar to Perencevitch *et al,* who found a 46 percent increase of *E. cloacae* in summer compared with winter. In our study, there was 43 percent increase in months with temperatures ≥20°C (compared to months <5°C). There was a 21 percent increase for *A. baumannii* and 37 percent respectively and a 12 percent increase for *E. coli* and 5 percent respectively. Perencevitch *et al* could not show a seasonal variation for *S. aureus*, but found that *Enterococcus* species were 9 percent less frequent in summer than in winter. The authors concluded that the different findings for Gram-positive and Gram-negative pathogens reduced the likelihood for confounding variables such as hospital-related factors, and underline the assumption that there are mechanistically different reasons for summer peaking only by Gram-negatives. In an other study, 211,697 blood stream infections were analysed in 132 U.S. hospitals over 8 years. They looked at the monthly outdoor temperature and showed that they were associated with substantially increased frequency of bloodstream infections among important Gram-negative bacteria [8]. These results are in line with our findings: an increase in monthly temperature of 5.6°C corresponded to independent increase in *A. baumannii* by 10.8% versus 8% our study, *E. coli* 3.5 versus 1%, *K. pneumonia* 8 versus 5% and *P. aeruginosa* 7.5 versus 3%. However, they did not investigate *Serratia, Stenotrophomonas, Enterobacter and Citrobacter* spp.

Our analysis considered hospital-related confounding parameters as well (long-term trends, number of pathogens in the previous month, and structural parameters, among others).

Other studies did not see seasonal variation in bloodstream infections (BSI) caused by *Enterobacter or Klebsiella.* These studies were limited by a very low number of pathogens and focus on one single pathogen, however [24], [25]. Anderson *et al*. investigated whether *K. pneumoniae, Enterobacter* or *Serratia* species had higher incidences during the warmest month of the year in four hospitals on four continents [26]. They found that increasing environmental temperature was independently associated with an increased incidence of *K. pneumoniae* BSI but not for *Serratia* or *Enterobacter* BSI. The authors explained their findings by the fact that *K. pneumoniae* is the most heat tolerant of all enteric pathogens and that *Klebsiella* survives better at higher humidity.

In contrast with Anderson *et al.*, our study revealed a linear and prominent temperature dependency of *E. cloacae,* We also found no seasonality for *S. marcescens*, which is in accordance with their findings. It remains unclear why *S. marcescens* seems to differ from other Gram-negatives. *S. marcescens* can also survive and grow under extreme conditions but the epidemiology is somewhat different from other Enterobactereaceae: *Serratia* appears to be less likely to colonize the gastrointestinal tract and more likely to colonize the respiratory or urinary tracts of hospitalized patients [27] [28]. The review by Leekha et al. on the seasonality of S. aureus showed that some studies could show an increase in incidence with increasing temperature but not an increase in colonizations. Leekha et al. determined, however, that there are important gaps in the literature on the seasonality of S. aureus infections, the most evident of which being a lack of appropriate statistical methodology (i.e. use of time-series analysis or time series regression models) for seasonality assessment, or the studies’ being performed in a single center and/or a single year [29]. By contrast, our study showed a small but significant decrease of S. aureus isolates of 1% (infections and colonizations) with a temperature increase of 5°C. In contrast to other pathogens with seasonal effects, this is of little clinical relevance.

Generally, an improved understanding of the forces driving seasonal physiologic changes and endogenous biologic rhythms may uncover unimagined opportunities for disease prevention. However, it was beyond the scope of our study to investigate physiology and susceptibility of Gram-positive and Gram-negative pathogens in different seasons. Regardless of the mechanism responsible for infection, our multicenter data have contributed to the emerging evidence for the seasonality of Gram-negative bacteria, and might have an impact for infection control, therapy and future study designs. Estimating the magnitude of the impact of those parameters provides more detailed information with the aim to prevent infections. The knowledge that Gram-negative infections were on average 15 percent more likely to occur in summer compared with winter could help in the selection of optimal empirical antibiotic therapy. Studies should consider seasonality as additional parameter. For example, studies – even randomized controlled trials – analyzing infections caused by Gram-negatives in summer versus in winter could considerably bias the results if they do not control for seasonality.

The strengths of our study are the multicenter design with 73 ICUs in 41 hospitals and 34 different cities in Germany, the inclusion of 13 Gram-positive and Gram-negative pathogens (n = 188,949 non-duplicate bacterial cultures), the relatively long study period of 12 years and the consideration of all isolates, *i.e*. not only isolates from blood culture. Additionally, our time-series regression models took cluster effects within an ICU into account, and considered different confounding parameters and adjusted for severity of illness of patients (length of stay and device use within the ICU).

Our study has also several limitations. First, all isolates were included and we could not differentiate by localization, origin (nosocomial or not regarding hospital and/or ICU), colonization or infection. Second, conclusions from our study may not be generalized for other settings because we analyzed only adult ICU patients. Third, our epidemiological data were obtained from an ongoing surveillance system which is ward-based, not patient-based, therefore making patient-specific analyses impossible. Forth, we could not exclude pathogens from outbreaks or screening which might have inflated the number of pathogens isolated.

It is interesting to speculate that seasonal variations in physiologic parameters might underlie the distinct seasonal variation of Gram-positive or Gram-negative pathogens by altering the susceptibility of host cells to infection. Even if the underlying mechanisms are not yet clear, the temperature-dependent seasonality of Gram-positive or Gram-negative pathogens has implications for infection control (surveillance, choice of empirical antibiotic treatment) and study design.
